# Dibromido(2,9-dimethyl-1,10-phenanthroline-κ^2^ *N*,*N*′)(dimethyl sulfoxide-κ*O*)cadmium

**DOI:** 10.1107/S1600536812050106

**Published:** 2012-12-12

**Authors:** Khadijeh Moghanlou

**Affiliations:** a Department of Chemistry, North Tehran Branch, Islamic Azad University, Tehran, Iran

## Abstract

In the mol­ecule of the title compound, [CdBr_2_(C_14_H_12_N_2_)(C_2_H_6_OS)], the Cd^II^ atom is five-coordinated in a distorted trigonal–bipyramidal configuration by two N atoms from a 2,9-dimethyl-1,10-phenanthroline ligand, one O atom from a dimethyl sulfoxide ligand and two Br atoms. In the crystal, π–π contacts between the pyridine and benzene rings [centroid–centroid distances = 3.710 (5), 3.711 (6) and 3.627 (5) Å] stabilize the structure.

## Related literature

For related structures, see: Akbarzadeh Torbati *et al.* (2010 ▶); Alizadeh *et al.* (2009 ▶); Armentano *et al.* (2006 ▶); Ding *et al.* (2006 ▶); Fanizzi *et al.* (1991 ▶); Lemoine *et al.* (2003 ▶); Robinson & Sinn (1975 ▶).
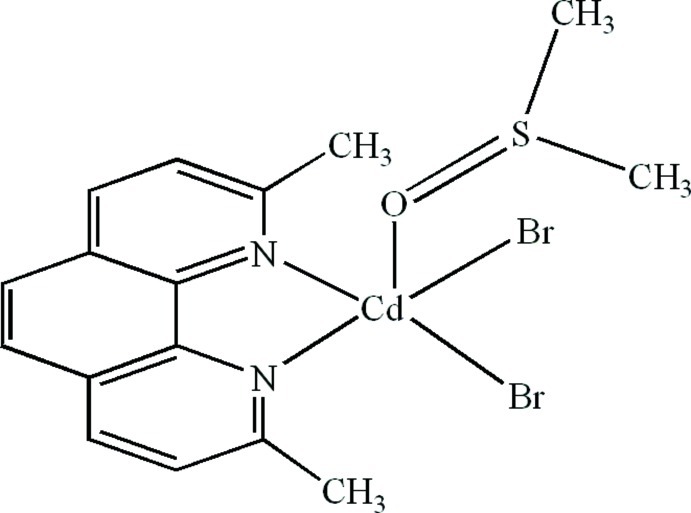


## Experimental

### 

#### Crystal data

[CdBr_2_(C_14_H_12_N_2_)(C_2_H_6_OS)]
*M* *_r_* = 558.60
Monoclinic, 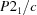

*a* = 8.1468 (9) Å
*b* = 17.3814 (15) Å
*c* = 13.6369 (13) Å
β = 95.724 (9)°
*V* = 1921.4 (3) Å^3^
*Z* = 4
Mo *K*α radiation
μ = 5.42 mm^−1^
*T* = 298 K
0.42 × 0.22 × 0.17 mm

#### Data collection

Bruker APEXII CCD diffractometer
Absorption correction: multi-scan (*SADABS*; Bruker, 2001 ▶) *T*_min_ = 0.222, *T*_max_ = 0.325
15831 measured reflections
3766 independent reflections
2196 reflections with *I* > 2σ(*I*)
*R*_int_ = 0.108

#### Refinement

*R*[*F* ^2^ > 2σ(*F* ^2^)] = 0.057
*wR*(*F* ^2^) = 0.127
*S* = 0.94
3766 reflections
208 parameters
H-atom parameters constrained
Δρ_max_ = 1.02 e Å^−3^
Δρ_min_ = −1.06 e Å^−3^

### 

Data collection: *APEX2* (Bruker, 2007 ▶); cell refinement: *SAINT* (Bruker, 2007 ▶); data reduction: *SAINT*; program(s) used to solve structure: *SHELXS97* (Sheldrick, 2008 ▶); program(s) used to refine structure: *SHELXL97* (Sheldrick, 2008 ▶); molecular graphics: *ORTEP-3* (Farrugia, 2012 ▶) and *Mercury* (Macrae *et al.*, 2006 ▶); software used to prepare material for publication: *SHELXL97*.

## Supplementary Material

Crystal structure: contains datablock(s) I, global. DOI: 10.1107/S1600536812050106/hy2608sup1.cif

Structure factors: contains datablock(s) I. DOI: 10.1107/S1600536812050106/hy2608Isup2.hkl

Additional supplementary materials:  crystallographic information; 3D view; checkCIF report

## Figures and Tables

**Table 1:** Selected bond lengths (Å)

Cd1—N1	2.386 (6)
Cd1—N2	2.331 (6)
Cd1—O1	2.361 (6)
Cd1—Br1	2.5483 (11)
Cd1—Br2	2.6335 (11)

**Fig. 1.**
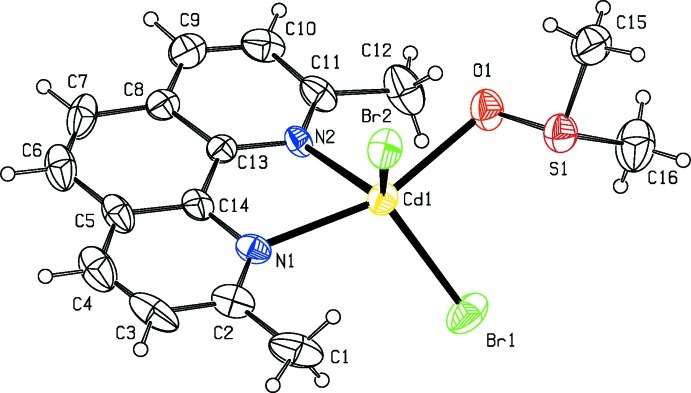
The molecular structure of the title compound. Displacement ellipsoids are drawn at the 30% probability level.

**Fig. 2.**
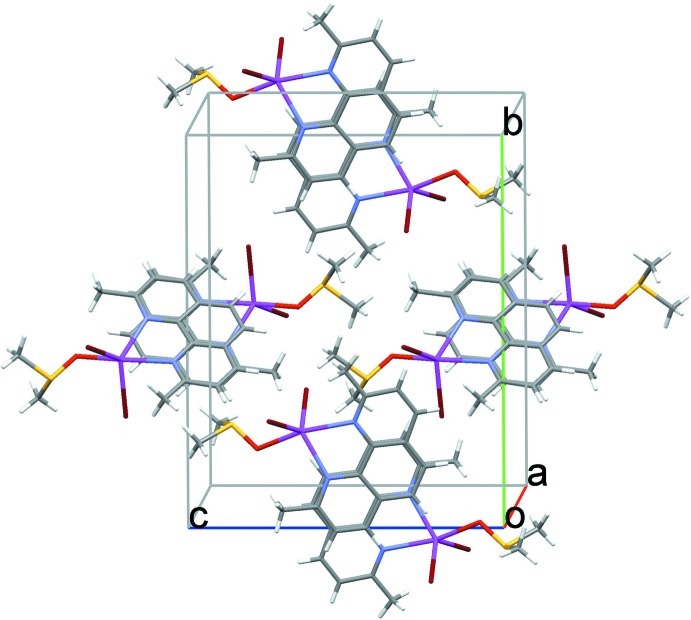
Crystal packing diagram for the title compound.

## supplementary crystallographic information

### Comment

2,9-Dimethyl-1,10-phenanthroline (Me_2_phen) is a good bidentate ligand, and numerous complexes with Me_2_phen have been prepared, such as that of mercury (Alizadeh *et al.*, 2009), iron (Armentano *et al.*, 2006), copper (Lemoine *et al.*, 2003), nickel (Ding *et al.*, 2006), gold (Robinson & Sinn, 1975), platinum (Fanizzi *et al.*, 1991) and cobalt (Akbarzadeh Torbati *et al.*, 2010). Here, we report the synthesis and structure of the title compound.

In the title compound (Fig. 1), the Cd^II^ atom is five-coordinated in a distorted trigonal-bipyramidal configuration by two N atoms from a 2,9-dimethyl-1,10-phenanthroline ligand, one O atom from a dimethyl sulfoxide ligand and two Br atoms (Table 1). In the crystal, π–π contacts between the pyridine and benzene rings, *Cg*2···*Cg*3^i^, *Cg*2···*Cg*4^i^ and *Cg*3···*Cg*4^ii^ [symmetry codes: (i) -x, 1-y, 2-z; (ii) 1-x, 1-y, 2-z, *Cg*2, *Cg*3 and *Cg*4 are the centroids of the N1/C2–C5/C14, N2/C8–C11/C13 and C5–C8/C13/C14 rings, respectively], with centroid–centroid distances of 3.710 (5), 3.711 (6) and 3.627 (5) Å, stabilize the structure (Fig. 2).

### Experimental

For the preparation of the title compound, a solution of 2,9-dimethyl-1,10-phenanthroline (0.42 g, 2.00 mmol) in methanol (15 ml) was added to a solution of CdBr_2_.4H_2_O, (0.69 g, 2.00 mmol) in methanol (15 ml) at room temperature. Crystals suitable for X-ray diffraction experiment were obtained by methanol diffusion into a colorless solution in DMSO after five days (yield: 0.85 g, 76.1%).

### Refinement

H atoms were positioned geometrically and refined as riding atoms, with C—H = 0.93 (CH) and 0.96 (CH_3_) Å and with *U*_iso_(H) = 1.2*U*_eq_(C).

### Crystal data

**Table:**

[CdBr_2_(C_14_H_12_N_2_)(C_2_H_6_OS)]	*F*(000) = 1080
*M**_r_* = 558.60	*D*_x_ = 1.936 Mg m^−^^3^
Monoclinic, *P*2_1_/*c*	Mo *K*α radiation, λ = 0.71073 Å
Hall symbol: -P 2ybc	Cell parameters from 15831 reflections
*a* = 8.1468 (9) Å	θ = 1.9–26.0°
*b* = 17.3814 (15) Å	µ = 5.42 mm^−^^1^
*c* = 13.6369 (13) Å	*T* = 298 K
β = 95.724 (9)°	Block, colorless
*V* = 1921.4 (3) Å^3^	0.42 × 0.22 × 0.17 mm
*Z* = 4	

### Data collection

**Table:**

Bruker APEXII CCD diffractometer	3766 independent reflections
Radiation source: fine-focus sealed tube	2196 reflections with *I* > 2σ(*I*)
Graphite monochromator	*R*_int_ = 0.108
φ and ω scans	θ_max_ = 26.0°, θ_min_ = 1.9°
Absorption correction: multi-scan (*SADABS*; Bruker, 2001)	*h* = −10→10
*T*_min_ = 0.222, *T*_max_ = 0.325	*k* = −21→21
15831 measured reflections	*l* = −15→16

### Refinement

**Table:**

Refinement on *F*^2^	Primary atom site location: structure-invariant direct methods
Least-squares matrix: full	Secondary atom site location: difference Fourier map
*R*[*F*^2^ > 2σ(*F*^2^)] = 0.057	Hydrogen site location: inferred from neighbouring sites
*wR*(*F*^2^) = 0.127	H-atom parameters constrained
*S* = 0.94	*w* = 1/[σ^2^(*F*_o_^2^) + (0.062*P*)^2^] where *P* = (*F*_o_^2^ + 2*F*_c_^2^)/3
3766 reflections	(Δ/σ)_max_ = 0.004
208 parameters	Δρ_max_ = 1.02 e Å^−^^3^
0 restraints	Δρ_min_ = −1.06 e Å^−^^3^

### Special details

**Table:**

Geometry. All e.s.d.'s (except the e.s.d. in the dihedral angle between two l.s. planes) are estimated using the full covariance matrix. The cell e.s.d.'s are taken into account individually in the estimation of e.s.d.'s in distances, angles and torsion angles; correlations between e.s.d.'s in cell parameters are only used when they are defined by crystal symmetry. An approximate (isotropic) treatment of cell e.s.d.'s is used for estimating e.s.d.'s involving l.s. planes.
Refinement. Refinement of *F*^2^ against ALL reflections. The weighted *R*-factor *wR* and goodness of fit *S* are based on *F*^2^, conventional *R*-factors *R* are based on *F*, with *F* set to zero for negative *F*^2^. The threshold expression of *F*^2^ > σ(*F*^2^) is used only for calculating *R*-factors(gt) *etc*. and is not relevant to the choice of reflections for refinement. *R*-factors based on *F*^2^ are statistically about twice as large as those based on *F*, and *R*- factors based on ALL data will be even larger.

### Fractional atomic coordinates and isotropic or equivalent isotropic displacement parameters (Å^2^)

**Table:**

	*x*	*y*	*z*	*U*_iso_*/*U*_eq_	
C1	−0.0593 (14)	0.2673 (6)	0.8844 (10)	0.096 (4)	
H1A	0.0219	0.2463	0.8456	0.115*	
H1B	−0.1421	0.2938	0.8423	0.115*	
H1C	−0.1097	0.2263	0.9180	0.115*	
C2	0.0215 (11)	0.3221 (5)	0.9583 (8)	0.064 (3)	
C3	0.0114 (13)	0.3105 (7)	1.0594 (10)	0.085 (4)	
H3	−0.0431	0.2677	1.0810	0.102*	
C4	0.0811 (14)	0.3619 (8)	1.1251 (9)	0.085 (3)	
H4	0.0748	0.3545	1.1921	0.102*	
C5	0.1625 (11)	0.4262 (6)	1.0924 (7)	0.062 (2)	
C6	0.2357 (14)	0.4818 (7)	1.1589 (7)	0.075 (3)	
H6	0.2307	0.4759	1.2263	0.090*	
C7	0.3105 (13)	0.5414 (7)	1.1252 (7)	0.076 (3)	
H7	0.3584	0.5773	1.1699	0.091*	
C8	0.3216 (9)	0.5535 (5)	1.0220 (6)	0.051 (2)	
C9	0.3992 (11)	0.6171 (6)	0.9837 (8)	0.068 (3)	
H9	0.4516	0.6532	1.0264	0.082*	
C10	0.3987 (11)	0.6265 (5)	0.8869 (9)	0.070 (3)	
H10	0.4490	0.6692	0.8617	0.084*	
C11	0.3206 (11)	0.5706 (5)	0.8227 (7)	0.059 (2)	
C12	0.3144 (15)	0.5823 (6)	0.7120 (8)	0.089 (4)	
H12A	0.2016	0.5831	0.6839	0.107*	
H12B	0.3717	0.5410	0.6834	0.107*	
H12C	0.3660	0.6304	0.6987	0.107*	
C13	0.2514 (9)	0.4992 (4)	0.9535 (6)	0.0415 (18)	
C14	0.1699 (9)	0.4338 (5)	0.9909 (6)	0.048 (2)	
C15	0.5347 (12)	0.3392 (7)	0.5318 (8)	0.091 (4)	
H15A	0.5942	0.3188	0.5904	0.110*	
H15B	0.5888	0.3848	0.5118	0.110*	
H15C	0.5318	0.3015	0.4801	0.110*	
C16	0.2687 (17)	0.4141 (8)	0.4484 (8)	0.105 (4)	
H16A	0.3515	0.4513	0.4365	0.126*	
H16B	0.1670	0.4400	0.4568	0.126*	
H16C	0.2524	0.3796	0.3933	0.126*	
N1	0.0990 (8)	0.3812 (4)	0.9263 (5)	0.0505 (17)	
N2	0.2556 (7)	0.5071 (3)	0.8554 (4)	0.0434 (15)	
O1	0.3558 (8)	0.4221 (4)	0.6386 (4)	0.0707 (17)	
Cd1	0.18778 (7)	0.39536 (3)	0.76580 (4)	0.04942 (19)	
Br1	−0.07113 (13)	0.39818 (7)	0.64445 (8)	0.0862 (4)	
Br2	0.36845 (12)	0.27030 (5)	0.79524 (7)	0.0636 (3)	
S1	0.3331 (3)	0.36172 (15)	0.55550 (18)	0.0659 (6)	

### Atomic displacement parameters (Å^2^)

**Table:**

	*U*^11^	*U*^22^	*U*^33^	*U*^12^	*U*^13^	*U*^23^
C1	0.072 (7)	0.067 (7)	0.154 (12)	−0.016 (6)	0.041 (7)	0.003 (7)
C2	0.055 (5)	0.044 (5)	0.093 (8)	0.007 (4)	0.017 (5)	0.013 (5)
C3	0.070 (7)	0.075 (8)	0.118 (10)	0.024 (6)	0.052 (7)	0.047 (7)
C4	0.086 (8)	0.104 (9)	0.068 (7)	0.038 (7)	0.025 (6)	0.032 (7)
C5	0.062 (6)	0.075 (6)	0.052 (6)	0.029 (5)	0.021 (5)	0.019 (5)
C6	0.091 (8)	0.088 (8)	0.047 (6)	0.038 (7)	0.011 (5)	0.014 (6)
C7	0.081 (7)	0.093 (9)	0.050 (6)	0.038 (6)	−0.010 (5)	−0.025 (5)
C8	0.042 (4)	0.049 (5)	0.061 (6)	0.010 (4)	0.005 (4)	−0.013 (4)
C9	0.055 (5)	0.069 (7)	0.080 (7)	0.010 (5)	0.004 (5)	−0.028 (5)
C10	0.057 (6)	0.051 (6)	0.106 (9)	−0.001 (4)	0.025 (6)	−0.021 (5)
C11	0.064 (6)	0.043 (5)	0.076 (6)	0.007 (4)	0.033 (5)	−0.007 (4)
C12	0.140 (10)	0.049 (6)	0.087 (8)	−0.008 (6)	0.050 (8)	0.014 (5)
C13	0.035 (4)	0.042 (5)	0.048 (5)	0.009 (3)	0.004 (3)	−0.006 (3)
C14	0.037 (4)	0.055 (5)	0.052 (5)	0.023 (4)	0.011 (4)	0.002 (4)
C15	0.072 (7)	0.109 (10)	0.091 (8)	0.012 (6)	0.003 (6)	−0.037 (7)
C16	0.123 (10)	0.116 (11)	0.073 (8)	0.033 (8)	−0.006 (7)	0.006 (7)
N1	0.040 (4)	0.050 (4)	0.062 (5)	0.007 (3)	0.011 (3)	0.008 (3)
N2	0.049 (4)	0.041 (4)	0.043 (4)	0.001 (3)	0.018 (3)	−0.004 (3)
O1	0.090 (5)	0.066 (4)	0.058 (4)	−0.004 (3)	0.016 (3)	−0.011 (3)
Cd1	0.0506 (3)	0.0497 (3)	0.0470 (3)	0.0041 (3)	0.0002 (2)	−0.0050 (3)
Br1	0.0643 (6)	0.0998 (9)	0.0882 (8)	0.0266 (6)	−0.0238 (5)	−0.0290 (6)
Br2	0.0725 (6)	0.0576 (6)	0.0593 (6)	0.0198 (5)	−0.0008 (4)	−0.0025 (4)
S1	0.0765 (16)	0.0627 (15)	0.0587 (15)	−0.0006 (12)	0.0080 (12)	−0.0057 (11)

### Geometric parameters (Å, º)

**Table:**

C1—C2	1.491 (15)	C11—N2	1.321 (10)
C1—H1A	0.9600	C11—C12	1.519 (13)
C1—H1B	0.9600	C12—H12A	0.9600
C1—H1C	0.9600	C12—H12B	0.9600
C2—N1	1.304 (10)	C12—H12C	0.9600
C2—C3	1.404 (14)	C13—N2	1.349 (9)
C3—C4	1.349 (16)	C13—C14	1.435 (11)
C3—H3	0.9300	C14—N1	1.358 (11)
C4—C5	1.395 (15)	C15—S1	1.750 (10)
C4—H4	0.9300	C15—H15A	0.9600
C5—C14	1.398 (11)	C15—H15B	0.9600
C5—C6	1.415 (15)	C15—H15C	0.9600
C6—C7	1.309 (14)	C16—S1	1.757 (11)
C6—H6	0.9300	C16—H16A	0.9600
C7—C8	1.435 (13)	C16—H16B	0.9600
C7—H7	0.9300	C16—H16C	0.9600
C8—C9	1.399 (13)	Cd1—N1	2.386 (6)
C8—C13	1.409 (11)	Cd1—N2	2.331 (6)
C9—C10	1.330 (14)	O1—S1	1.542 (6)
C9—H9	0.9300	Cd1—O1	2.361 (6)
C10—C11	1.416 (13)	Cd1—Br1	2.5483 (11)
C10—H10	0.9300	Cd1—Br2	2.6335 (11)
			
C2—C1—H1A	109.5	H12A—C12—H12C	109.5
C2—C1—H1B	109.5	H12B—C12—H12C	109.5
H1A—C1—H1B	109.5	N2—C13—C8	122.7 (7)
C2—C1—H1C	109.5	N2—C13—C14	119.4 (7)
H1A—C1—H1C	109.5	C8—C13—C14	117.8 (7)
H1B—C1—H1C	109.5	N1—C14—C5	121.4 (8)
N1—C2—C3	121.3 (10)	N1—C14—C13	119.0 (7)
N1—C2—C1	118.3 (9)	C5—C14—C13	119.7 (8)
C3—C2—C1	120.4 (10)	S1—C15—H15A	109.5
C4—C3—C2	119.7 (10)	S1—C15—H15B	109.5
C4—C3—H3	120.2	H15A—C15—H15B	109.5
C2—C3—H3	120.2	S1—C15—H15C	109.5
C3—C4—C5	120.0 (10)	H15A—C15—H15C	109.5
C3—C4—H4	120.0	H15B—C15—H15C	109.5
C5—C4—H4	120.0	S1—C16—H16A	109.5
C4—C5—C14	117.5 (10)	S1—C16—H16B	109.5
C4—C5—C6	121.7 (10)	H16A—C16—H16B	109.5
C14—C5—C6	120.8 (9)	S1—C16—H16C	109.5
C7—C6—C5	119.8 (9)	H16A—C16—H16C	109.5
C7—C6—H6	120.1	H16B—C16—H16C	109.5
C5—C6—H6	120.1	C2—N1—C14	120.2 (8)
C6—C7—C8	122.5 (10)	C2—N1—Cd1	126.1 (6)
C6—C7—H7	118.7	C14—N1—Cd1	112.2 (5)
C8—C7—H7	118.7	C11—N2—C13	118.0 (7)
C9—C8—C13	116.8 (8)	C11—N2—Cd1	127.0 (5)
C9—C8—C7	123.8 (9)	C13—N2—Cd1	114.0 (5)
C13—C8—C7	119.4 (9)	S1—O1—Cd1	111.7 (3)
C10—C9—C8	120.7 (9)	N2—Cd1—O1	95.5 (2)
C10—C9—H9	119.6	N2—Cd1—N1	71.5 (2)
C8—C9—H9	119.6	O1—Cd1—N1	161.0 (2)
C9—C10—C11	119.0 (9)	N2—Cd1—Br1	117.50 (16)
C9—C10—H10	120.5	O1—Cd1—Br1	91.26 (17)
C11—C10—H10	120.5	N1—Cd1—Br1	106.91 (16)
N2—C11—C10	122.4 (9)	N2—Cd1—Br2	120.54 (16)
N2—C11—C12	118.2 (8)	O1—Cd1—Br2	85.32 (17)
C10—C11—C12	119.3 (9)	N1—Cd1—Br2	89.49 (15)
C11—C12—H12A	109.5	Br1—Cd1—Br2	121.92 (4)
C11—C12—H12B	109.5	O1—S1—C15	104.0 (5)
H12A—C12—H12B	109.5	O1—S1—C16	105.2 (5)
C11—C12—H12C	109.5	C15—S1—C16	99.8 (6)
			
N1—C2—C3—C4	0.9 (14)	C5—C14—N1—Cd1	−166.5 (6)
C1—C2—C3—C4	−178.1 (10)	C13—C14—N1—Cd1	14.6 (8)
C2—C3—C4—C5	0.1 (15)	C10—C11—N2—C13	6.0 (11)
C3—C4—C5—C14	−0.8 (14)	C12—C11—N2—C13	−175.7 (8)
C3—C4—C5—C6	179.4 (9)	C10—C11—N2—Cd1	−162.1 (6)
C4—C5—C6—C7	−179.8 (9)	C12—C11—N2—Cd1	16.2 (11)
C14—C5—C6—C7	0.4 (14)	C8—C13—N2—C11	−3.7 (10)
C5—C6—C7—C8	0.2 (15)	C14—C13—N2—C11	175.0 (7)
C6—C7—C8—C9	179.3 (9)	C8—C13—N2—Cd1	165.9 (5)
C6—C7—C8—C13	−0.7 (13)	C14—C13—N2—Cd1	−15.3 (8)
C13—C8—C9—C10	3.0 (12)	C11—N2—Cd1—O1	19.0 (7)
C7—C8—C9—C10	−177.0 (8)	C13—N2—Cd1—O1	−149.5 (5)
C8—C9—C10—C11	−1.0 (13)	C11—N2—Cd1—N1	−175.2 (7)
C9—C10—C11—N2	−3.7 (13)	C13—N2—Cd1—N1	16.3 (5)
C9—C10—C11—C12	177.9 (9)	C11—N2—Cd1—Br1	−75.4 (7)
C9—C8—C13—N2	−0.6 (11)	C13—N2—Cd1—Br1	116.1 (5)
C7—C8—C13—N2	179.4 (7)	C11—N2—Cd1—Br2	106.8 (7)
C9—C8—C13—C14	−179.5 (7)	C13—N2—Cd1—Br2	−61.7 (5)
C7—C8—C13—C14	0.5 (10)	S1—O1—Cd1—N2	−175.0 (4)
C4—C5—C14—N1	0.7 (11)	S1—O1—Cd1—N1	139.3 (6)
C6—C5—C14—N1	−179.5 (8)	S1—O1—Cd1—Br1	−57.2 (4)
C4—C5—C14—C13	179.6 (8)	S1—O1—Cd1—Br2	64.7 (4)
C6—C5—C14—C13	−0.6 (11)	C2—N1—Cd1—N2	178.3 (7)
N2—C13—C14—N1	0.2 (10)	C14—N1—Cd1—N2	−15.9 (5)
C8—C13—C14—N1	179.0 (6)	C2—N1—Cd1—O1	−133.1 (8)
N2—C13—C14—C5	−178.8 (7)	C14—N1—Cd1—O1	32.7 (10)
C8—C13—C14—C5	0.1 (10)	C2—N1—Cd1—Br1	64.2 (7)
C3—C2—N1—C14	−1.0 (12)	C14—N1—Cd1—Br1	−130.0 (5)
C1—C2—N1—C14	177.9 (8)	C2—N1—Cd1—Br2	−59.1 (7)
C3—C2—N1—Cd1	163.7 (6)	C14—N1—Cd1—Br2	106.7 (5)
C1—C2—N1—Cd1	−17.3 (11)	Cd1—O1—S1—C15	−134.0 (5)
C5—C14—N1—C2	0.2 (11)	Cd1—O1—S1—C16	121.6 (5)
C13—C14—N1—C2	−178.7 (7)		
